# Extensor mechanism reconstruction in the setting of infected total knee arthroplasty

**DOI:** 10.7150/jbji.47622

**Published:** 2020-06-12

**Authors:** Lucy Cogswell, Rob McCulloch

**Affiliations:** Nuffield Orthopaedic Centre, Oxford University Hospitals NHS Foundation Trust, UK

Infection in a total knee arthroplasty (TKA) is a devastating but rare complication with an incidence of roughly one percent 1. A timely and accurate diagnosis of periprosthetic joint infection (PJI) - thorough pre-operative planning and adherence to treatment principles, ideally within a multidisciplinary setting - are essential in successful management 2. The decision regarding whether the patient is appropriate for debridement, antibiotics and implant retention (DAIR), single stage or two stage revision is based on many factors including knowledge of the pathogen, component fixation, patient co-morbidities and the surrounding soft-tissue envelope 3. Success rates of a DAIR procedure vary between institutions from 31% to100% when using eradication of infection as the outcome measure 3. Key to the success of any revision procedure for PJI is adequate soft tissue cover and where necessary plastic surgical techniques to facilitate this.

Bringing healthy soft tissue into the wound releases tension in the skin around the knee, helps to fill dead space, and adds well-vascularised tissue which is postulated to improve antibiotic delivery to the wound 4. As yet there is no definite evidence that a muscle flap is superior to a skin and fascia flap in the face of infection. The proximity of the gastrocnemius muscle, its convenient anatomy in terms of length and vascular pedicle, and its expendability make it a frequent choice for a flap in this region 5-9. The majority of infected TKAs needing flap cover can be closed with gastrocnemius muscle flaps. In our personal series of forty-six flaps for infected TKA, only two needed flaps other than gastrocnemius: one due to the size of the defect, the second because gastrocnemius had already been used in a previous procedure. Both had free latissimus dorsi reconstructions (unpublished data).

It is unusual for the extensor mechanism to be deficient after infection; in our practice this situation more commonly arises after tumour resection from the proximal tibia 4. However, similar reconstructive principles can be applied. Whilst a muscle-alone reconstruction has been described for the patellar tendon using only the fascia around a muscle flap to bridge the tendon gap 10, 11, a common issue with all reconstructive techniques for a failed extensor mechanism in the context of revision arthroplasty or sarcoma is an extensor lag 12, 13.

The utilisation of a robust tissue such as a tendon to integrate into the extensor remnant helps to minimise this problem 12, 13. Due to the extensive soft tissue releases associated with revision surgeries often requiring constrained hinged implants, stiffness is not frequently a significant impediment to patient function and outcome 14. Vascularised, autologous tissue is preferable over alloplastic implants in the presence of infection 15. Around the knee, two of the most commonly described vascularised autologous tissue reconstructions are the gastrocnemius muscle with attached Achilles tendon, and a tube of fascia created as part of an anterolateral thigh (ALT) flap. However, any other expendable tendon or dense fascia with a known vascular pedicle can be used. Around the ankle, multiple flaps have been described to reconstruct the Achilles tendon, including local tissue 4 fascia lata 15, adductor magnus tendon 16, groin flaps 17 parascapular flaps 18 as well as the ALT flap 19, and all of these techniques could equally be applied to the knee.

In our unit, we routinely use the gastrocnemius muscle flap for extensor mechanism reconstruction. If a two-stage procedure is planned, the muscle flap is ideally performed at the first stage. After wound debridement and lavage, approximately 25% of the Achilles tendon is harvested in continuity with the medial head of gastrocnemius, based on the proximal neurovascular pedicle. The tendon is sharply dissected from the distal part of the muscle, keeping the proximal portion of tendon attached to the muscle. This dissection allows a degree of freedom in arranging the tendon and muscle in the recipient bed. The fibrous tissue on the anterior and posterior surfaces of the muscle is scored in a 'mango-cutting' technique. This increases the effective surface area of muscle and makes it more supple to conform to the recipient bed. The flap is tunnelled subcutaneously to the defect. The proximal end of the tendon is sutured to the residual extensor remnant. The tendon is then fed through a transverse cut in the gastrocnemius muscle belly. Its distal end is sutured on (i) to the tibial periosteum in the region of the tibial tuberosity and (ii) to any residual patellar ligament if present. During this procedure the knee is in extension. In the context of a proximal tibial replacement where the tibial tuberosity has been sacrificed, the tendon is attached to the fascia around tibialis anterior. The native knee skin and the muscle flap are arranged to completely cover the Achilles tendon. The lateral head of gastrocnemius is also added to the construct when needed for soft tissue cover. Any exposed muscle is covered with split thickness skin graft.

Postoperatively, we keep the knee in extension for 3 months, and then begin gradual flexion under the supervision of a physiotherapist, using a hinged knee brace to incrementally increase the range and allow the patient to mobilise locked in extension to minimise the risk of extensor lag.

In addition to a thorough and careful debridement, three elements of surgical practice help to preserve the soft tissue envelope. Firstly, in the first part of a two-stage procedure, a cement spacer is used which reflects the real dimensions of the planned implant. This avoids the skin contraction which can result from leaving an 'underfilled' joint. Secondly, anterior knee wounds are not left open to drain which avoids skin retraction. Thirdly, if the knee does need to be opened more than once, care is taken with wound closure to avoid the scenario where sutures cut through the skin as it swells, leading to a frayed skin margin, or even complete skin necrosis along the suture line.

In our practice, we rarely need to use free flaps to replace lost skin in infected total knee arthroplasty, and therefore gastrocnemius-based flaps form the majority of extensor mechanism reconstructions. Equally, on the rare cases when we do encounter a larger skin defect, it is important that as surgeons working in the field of infected TKA, plastic surgeons have the flexibility to reconstruct all defects, as the case series of Osinga et al. 20 so beautifully illustrates.
